# Prevalence of drug resistance associated mutations in *Plasmodium vivax* against sulphadoxine-pyrimethamine in southern Pakistan

**DOI:** 10.1186/1475-2875-12-261

**Published:** 2013-07-26

**Authors:** Afsheen Raza, Najia K Ghanchi, Muhammad Shahzeb Khan, Mohammad Asim Beg

**Affiliations:** 1 Department of Pathology and Microbiology, Aga Khan University, Stadium Road, PO Box 3500, 74800, Karachi, Pakistan; 2 Dow Medical College, Civil Hospital, 74800, Karachi, Pakistan

**Keywords:** *Plasmodium vivax*, Pakistan, Dihydrofolate reductase, Dihydroptereoate synthase, Single nucleotide polymorphisms

## Abstract

**Background:**

In Pakistan, *Plasmodium vivax* and *Plasmodium falciparum* co-exist and usage of sulphadoxine-pyrimethamine (SP) against *P. falciparum* exposes *P. vivax* to the drug leading to generation of resistant alleles. The main aim of this study was to investigate frequency distribution of drug resistance associated mutations in *pvdhfr*, *pvdhps* genes and provide baseline molecular epidemiological data on SP-associated resistance in *P. vivax* from southern Pakistan.

**Methods:**

From January 2008 to May 2009, a total of 150 samples were collected from patients tested slide-positive for *P. vivax*, at the Aga Khan University Hospital, Karachi, or its collection units located in Baluchistan and Sindh Province. Nested PCR using *pvdhfr* and *pvdhps* specific primers was performed for all samples.91.3% (137/150) of the samples were tested PCR positive of which 87.3% (131/137) were successfully sequenced. Sample sequencing data was analysed and compared against wild type reference sequences.

**Results:**

In *dhfr*, mutations were observed at codons F57L, S58R and S117N/T. Novel non-synonymous mutations were observed at codon positions N50I, G114R and E119K while a synonymous mutation was observed at codon position 69Y. In *dhps*, mutations were observed at codon position A383G and A553G while novel non-synonymous mutations were observed at codon positions S373T, E380K, P384L, N389T, V392D, T393P, D459A, M601I, A651D and A661V.

**Conclusion:**

This is the first report from southern Pakistan on SP resistance in clinical isolates of *P. vivax.* Results from this study confirm that diverse drug resistant alleles are circulating within this region.

## Background

*Plasmodium vivax* malaria is an important public health problem worldwide causing an estimated 80–215 million clinical infections annually. Geographically, it is found throughout Asia, South and Central America, Middle East and some parts of Africa [1].

**Figure 1:**
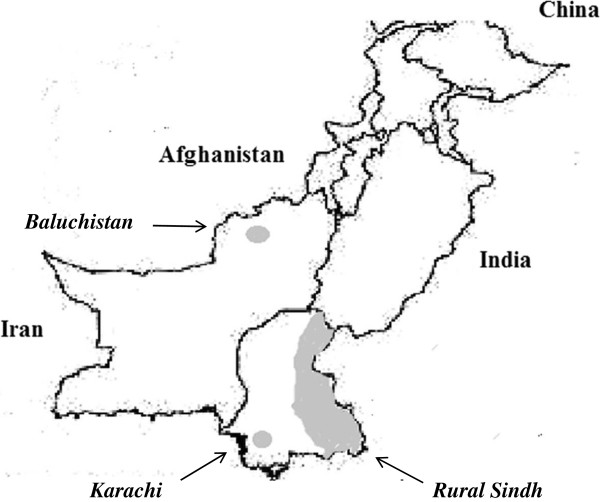
Map of Pakistan showing study sites.

Malaria is endemic throughout Pakistan with an estimated 4.5 million suspected cases reported by World Health Organization [2]. Southern Pakistan, where this study was conducted, comprises of moderate to high malaria endemicity areas with annual parasite incidences reported as high as 5.5/1,000 population [3] see Figure 1. Both *P. vivax* and *Plasmodium falciparum* co-exist, with *P. vivax* being the major contributor (70%) of malaria burden in all areas [2].

In Pakistan, the treatment guidelines recommended by National Malaria Control Programme (MCP) until 2007 was chloroquine (CQ) as the first-line and sulphadoxine-pyrimethamine (SP) as the second-line treatment of uncomplicated *P. falciparum* malaria. With reports of CQ resistance, the treatment policy was changed in 2008, with the combination of artesunate (AS) plus sulphadoxine-pyrimethamine against uncomplicated *P. falciparum* and chloroquine plus primaquine recommended as the first-line treatment for *P. vivax* malaria, respectively [4]. Though recommended, implementation of treatment guidelines is non-existent with studies documenting only 1% of patients receiving combination therapies [2,5]. Furthermore, with co-existence of both species in significant proportions, issues of misdiagnosis due to sub-microscopic infections in association with *P. falciparum*[6] and inappropriate treatment with mono-therapies exist in the public sector [7]. The consequence of such approach may have implications on *P. vivax* control in the region since mono-therapy may induce drug pressure on *P. vivax* resistance associated genes and trigger the selection of resistant alleles. As yet, limited data is available from Pakistan on the prevalence of resistance in *P. vivax.*

Molecular and epidemiological studies conducted in various malaria-endemic regions have shown that therapeutic targets of SP in both *P. falciparum* and *P. vivax* are *dihydrofolate reductase* (*dhfr*) and *dihydroptereoate synthase* (*dhps*) enzymes. *Dhfr* codons for pyrimethamine (PYR) interaction have been identified as I13, P33, F57, S58, T61, S117 and I173 while *dhps* codons S382, A383, K512, A553 and V585 are sulphadoxine (SPX) binding codons [8]. Single nucleotide polymorphism (SNP) in SPX and PYR binding sites is the mechanism employed by *Plasmodium* species to survive drug pressure and transmit effectively. Briefly, accumulation of SNPs lead to changes in amino acid residues at key codon positions resulting in alteration of drug interaction sites, thus preventing drugs binding to enzyme active sites [9,10]. Changes in amino acid residues commonly associated with SPX and PYR resistance are I13**L**, P33**L**, F57**L/I**, S58**R**, T61**M**, S117**N/T**, I173**L/F** and 382**A**/**C**, 383**G**, 512**M/T/E**, 553**G**/**C**, V585(any non-synonymous amino acid change) [8]. Furthermore, SNPs leading to insertion/deletion of amino acids in known repeat regions of *dhfr* (GGDNTS/TH/ADK) and *dhps* (GEAKLTN/G/A/G/A/G/GDAKLTN/S/A) result in generation of tandem repeat variant types (TRV types). These TRV types have been used in studies for genotyping of *dhfr* and *dhps* alleles [11,12]. Some studies have also suggested possible association of TRV types with mutant alleles and disease severity [11,13,14].

Data on SP-resistant alleles have been reported from various malaria-endemic regions, such as Thailand, Indonesia, Papua New Guinea, Madagascar, Iran, India and Afghanistan. However, limited studies on SP resistance and cure rates have been documented from northern Pakistan [6,15,16] where SP is rendered effective against *P. vivax*. However, data on SP resistance from southern Pakistan is lacking. Therefore, the aim of this study was to investigate frequency distribution of SP resistance associated mutations in *pvdhfr* and *pvdhps* genes in clinical isolates from southern Pakistan so that baseline molecular epidemiological data on extent of SP resistance could be documented.

## Methods

### Study design, settings and ethical considerations

A descriptive study to determine the prevalence of drug resistance associated single nucleotide polymorphisms in *P. vivax* clinical isolates was carried out between January 2008 and May 2009. A total of 150 patients, presenting with microscopy-confirmed, asexual *P. vivax* mono-infection at Aga Khan University Hospital, Karachi or its collection units located in Sindh and Baluchistan provinces were enrolled in the study. The study was approved by the Ethical Review Committee of Aga Khan University Hospital and conducted in accordance with the Good Clinical Practice of Declaration of Helsinki [17]. Informed consent was obtained from enrolled patients or in case of children from their parents/legal guardians.

### Blood collection and microscopy

Approximately 2 ml of intravenous blood sample in EDTA tube was collected. Initial presence of malaria parasites was established by Leishman’s staining while further species identification was determined by Giemsa staining of thick and thin blood smears [18]. The remaining blood was stored at -80°C until further analysis.

### DNA extraction and amplification

DNA was extracted from 200 μl of whole blood using QiAamp DNA Mini Kit according to manufacturer’s instructions (QIAGEN, USA). *Plasmodium vivax dhfr* and *dhps* genes were amplified using nested PCR methodology as described previously [19,20]. Analysis of PCR products was done by gel electrophoresis. Briefly, amplified product was loaded on 1.5% agarose gels alongside 100 bp molecular weight markers, separated in Tris borate (TBE) buffer by electrophoresis, stained with ethidium bromide and visualized under UV transilluminator (Gel Doc, Bio-Rad, Hercules, USA).

### Sequencing and similarity analysis

PCR-positive amplified products of *dhfr* and *dhps* were purified using QIA quick Qiagen PCR purification kit (QIAGEN, USA). Direct sequencing of purified products in both directions was performed commercially (Macrogen, Korea). Nucleotide and amino acid sequences were compared with wild type *dhfr* and *dhps* reference sequences using pair wise sequence similarity analysis software BLAST. Gen Bank accession numbers of wild type sequences used for comparison are ARI/Pakistan X98123 for *dhfr* and AY186730 for *dhps.*

### Data interpretation

Samples were interpreted as wild type (WT) if no mutations were observed in the entire *dhfr* and *dhps* coding region. Single, two, three or four mutations observed either in *dhfr*, *dhps* or both coding regions was designated as single, double, triple and quadruple mutations respectively (SM, DM, TM and QM). The *dhfr* and *dhps* TRV types were categorized as Type A-C and Type 1a-6a on the basis of insertion/deletion of amino acids at the repeat codon position 88–106 of *dhfr* and 603–665 of *dhps*.

### Statistical analysis

Data were entered in Microsoft Excel and exported to SPSS version 19.0 for analysis. Arithmetical means and medians were calculated where applicable, for all continuous baseline demographic variables. Allele proportions were calculated as the number carrying a certain allele divided by the number of samples with positive PCR outcome.

## Results

### Baseline demographic data

A total of 150 patients with microscopy-confirmed *P. vivax* mono-infection were enrolled from Karachi, Baluchistan and Rural Sindh. Out of these, 137 (91.3%) were PCR positive for both *dhfr* and *dhps* and were thus selected for sequencing. Sequencing was successful in 131 (87.3%) samples of which 98 were from Karachi, 13 from Baluchistan and 20 from Rural Sindh.

### Distribution of *pvdhfr* and *pvdhps* mutations

Sequence analysis revealed mutations in *dhfr* codons F57**L**, S58**R** and S117**N**/**T** while no mutation was observed at P33, T61 and I173. Novel non-synonymous mutations were observed at codon positions N50**I**, G114**R** and E119**K** while synonymous mutation was observed at codon position 69Y. In *dhps*, mutations at the SPX binding sites was observed at codon position A383**G** and A553**G** while non-synonymous mutations were observed at codon positions S373**T**, E380**K**, P384**L**, N389**T**, V392**D**, T393**P**, D459**A**, M601**I**, A651**D** and A661**V**.

Haplotyping analysis revealed identification of nine *pvdhfr-pvdhps* types in the study areas (Table 1). Of these, pure WT isolates were observed in 38.9% (51/131) of the total isolates and in all the three sampling sites. Single mutation in *dhfr* was observed in 19.8% of the total isolates at codon position 58**R** and 117**N,** respectively. Of these, 58**R** were detected in Karachi isolates only while 117**N** was observed in all sampling sites in frequencies of 17.3% (Karachi), 15% (Sindh) and 30.8% (Baluchistan), respectively. Double mutation in *dhfr* was observed in 27.4% of the total isolates in a combination of 58**R**/ 117**N/T** and 57**L**/ 58**R,** respectively. Of these, 58**R**/ 117**N/T** were found to be the frequently isolated DM in all the study sites (25.2%). DM 57**L**/ 58**R** was observed in Karachi and Sindh only.

**Table 1:** **Frequency distribution of *pvdhfr-pvdhps *haplotypes in *P. vivax *isolates from southern Pakistan**

**pvdhfr-pvdhps haplotypes**	**Mutations**	**Karachi**	**Sindh**	**Baluchistan**	**Total (%)**
IPFSTSI-AA	0	40 (40.8)	8 (40.0)	3 (23.1)	51 (38.9)
IPFST**N/T**I-AA	1	17 (17.3)	3 (15.0)	4 (30.8)	24 (18.3)
IPF**R**TSI-AA	1	2 (2.04)	-	-	2 (1.53)
IPFSTSI-**G**A	1	7 (7.14)	2 (10.0)	-	9 (6.87)
IPF**R**T**N/T**I -AA	2	23 (23.5)	5 (25.0)	5 (38.5)	33 (25.2)
IP**LR**TSI-AA	2	2 (2.04)	1 (18.9)	-	3 (2.29)
IPFST**N/T**I-**G**A	2	3 (3.06)	-	-	3 (2.29)
IPF**R**T**N**/**T**I-**G**A	3	1 (1.02)	-	-	1 (0.76)
IPF**R**T**N/T**I-**GG**	4	-	-	1 (7.7)	1 (0.76)

pvdhfr: I13**L** ,P33**L**,F57**L**,S58**R**,T61**M**,S117**N/T**,I173**L.**

pvdhps**:** A383**G**,A553**G**.

Bold face represents change in amino acid.

In *dhps*, single mutant 383**G** was observed in frequencies of 7.4 and 10% in Karachi and rural Sindh respectively. Double, triple and quadruple alleles in combination with *dhfr* alleles (117**N/T**/383**G**, 58**R**/117**N**/**T**/383**G**, 58**R**/117**N/T**/3838**G**/553**G**) were observed. DM and TM were observed in low frequencies and were isolated from Karachi only while QM was observed in one isolate from Baluchistan.

Of the other non-synonymous mutations in *dhfr*, SM was observed in 114**R** and 119**K**. DM was observed in combination of 50**I**/119**K**, 114**R/**119**K**, 119**K**/117**N** and 50**I**/117**N**. DM found predominantly in all the sampling sites was 50**I**/117**N** observed in proportions of 5.1% in Karachi, 7.7% in Baluchistan and 15% in Sindh. All other mutant alleles, including distinctive 117**T**, were observed infrequently in the three sampling areas.

Other non-synonomous mutations observed in *dhps* were S373**T**, E380**K**, P384**L**, N389**T**, V392**D**, T393**P**, D459**A**, M601**I**, A651**D** and A661**V**. Of these, with the exception of 373 **T**, 601**I**, 651**D** and 661 **V**, all other were observed in Karachi only while these mutant alleles were observed in low frequencies in all the sampling sites.

### Distribution of *pvdhfr* and *pvdhps* tandem repeat variant types

TRV types were classified as Type A-C based on deletion/insertion of six amino acids at codon position 88–106 (Table 2). Type B was found to be the predominant TRV with 94.6% (124/131) of the isolates from all sampling sites exhibiting this type. Type A and C were observed in 3.8% and 1.5% of the isolates from Karachi only (Table 3). Type B was found to be commonly associated with WT, single and double *dhfr* (117**N/T** and 58**R**/117**N/T**) while Type A and C were associated with wild type alleles.

**Table 2:** **Tandem repeat variants types of *dhfr***

**Types**	**No. of**	**Amino acid polymorphisms (Insertions/**
	**repeats**	**deletions)**
A	4	GGDNTS GGDNTH GGDNTH GGDNAD
B	3	GGDNTS GGDNTH GGDNAD
C	2	------ GGDNTH GGDNAD

Underline represents insertion and ------ represents deletion of amino acid residues at the respective codon position.

**Table 3:** Distribution of dhfr tandem repeat types in pvdhfr-pvdhps haplotypes

**Types**				** pvdhfr**				**pvdhps**		**Total**
	I13**L**	P33**L**	F57**L**	S58**R**	T61**M**	S117**N/T**	I173**L**	A383**G**	A553**G**	**n = 131 (%)**
A	I	P	F	S	T	S	I	A	A	4 (3.05)
	I	P	F	S	T	**N/T**	I	A	A	1 (0.76)
B	I	P	F	S	T	S	I	A	A	45 (34.3)
	I	P	F	S	T	**N/T**	I	A	A	23 (17.5)
	I	P	F	**R**	T	S	I	A	A	2 (1.52)
	I	P	F	**R**	T	**N/T**	I	A	A	33 (25.2)
	I	P	**L**	**R**	T	S	I	A	A	3 (2.32)
	I	P	F	S	T	S	I	**G**	A	9 (6.87)
	I	P	F	**R**	T	S	I	**G**	A	1 (0.76)
	I	P	F	S	T	**N/T**	I	**G**	A	3 (2.32)
	I	P	F	**R**	T	**N/T**	I	**G**	A	4 (3.05)
	I	P	F	**R**	T	**N/T**	I	**G**	**G**	1 (0.76)
C	I	P	F	S	T	S	I	A	A	2 (1.52)

In *dhps,* six TRV types, designated as Type 1–6 and sub-typed as a, b, c, and d, were identified and categorized depending on the insertion and deletion of amino acids at various codon positions in the repeat region (Table 4). Type 3 (subtype 3a) consisting of seven tandem repeats and amino acids deleted at codon positions 631–644 was found to be the predominant with 51.9% of the isolates from all study sites exhibiting this type (Table 5). This type was found to be associated with WT, SM, DM, TM and QM haplotypes. Distinct type 6 comprising of 11 repeats was observed in three isolates and was found associated with TM alleles.

**Table 4:** **Tandem repeat variant types in *dhps***

**Types**	**No. of repeats**	**Amino acid polymorphisms**
		**(Insertions/deletions)**
**1a**	5	GEAKLTN GEGKLTN ------- ------- ------- ------- GDAKLTN GDSKLTN GEAKLTN
**1b**	5	GEAKLTN GEGKLTN ------- ------- ------- ------- GDAKLTN GDSKLTN GE**V**KLTNK
**2a**	6	GEAKLTN GEGKLTN GEAKLTN GEGKLTN ------- ------- ------- GDSKLTN GEAKLTN
**2b**	6	GEAKLTN GEGKLTN GEAKLTN GEGKLTN ------- ------- GDAKLTN------- GEAKLTN
**2c**	6	GEAKLTN GEGKLTN GEAKLTN GEGKLTN ------- ------- GDAKLTN GDSKLTN -------
**2d**	6	GEAKLTN ------- GEAKLTN GEGKLTN ------- ------- GDAKLTN GDSKLTN GE**V**KLTN
**3a**	7	GEAKLTN GEGKLTN GEAKLTN GEGKLTN ------- ------- GDAKLTN GDSKLTN GEAKLTN
**3b**	7	GEAKLTN GEGKLTN GEAKLTN GEGKLTN GEAKLTN ------- ------- GDSKLTN GEAKLTN
**3c**	7	GEAKLTN GEGKLTN GEAKLTN GEGKLTN ------ ------ GDAKLT**D** GDSKLTN GEAKLTN
**4a**	8	GEAKLTN GEGKLTN GEAKLTN GEGKLTN GEAKLTN GEGKLTN GDAKLTN ------- GEAKLTN
**4b**	8	GEAKLTN GEGKLTN GEAKLTN GEGKLTN GEAKLTN GEGKLTN GDAKLTN GDSKLTN -------
**4c**	8	GEAKLTN GEGKLTN GEAKLTN GEGKLTN ------ ------ GDAKLTN GDSKLTN GDSKLTN GEAKLTN
**5a**	9	GEAKLTN GEGKLTN GEAKLTN GEGKLTN GEAKLTN GEGKLTN GDAKLTN GDSKLTN GEAKLTN
**6a**	11	GEAKLTN GEGKLTN GEAKLTN GEGKLTN GEAKLTN GEGKLTN GEAKLTN GEGKLTN GDAKLTN GDSKLTN GEAKLTN

Bold face represents a novel substitution of amino acid residue at the designated codon position.

Underline represents insertion and ------ represents deletion of amino acid residues at the respective codon position.

**Table 5:** Distribution of dhps tandem repeat types in pvdhfr-pvdhps haplotypes

**Types**				** pvdhfr**				**pvdhps**		**Total**
	13I**L**	P33**L**	F57**L**	S58**R**	T61**M**	S117**N/T**	I173**L**	A383**G**	A553**G**	**n = 131 (%)**
**1**	I	P	F	S	T	S	I	A	A	
	I	P	F	S	T	**N/T**	I	A	A	20 (15.3)
	I	P	F	**R**	T	**N/T**	I	A	A	
	I	P	F	S	T	S	I	**G**	A	
**2**	I	P	F	S	T	S	I	A	A	
	I	P	F	S	T	**N/T**	I	A	A	
	I	P	F	**R**	T	S	I	A	A	
	I	P	F	**R**	T	**N/T**	I	A	A	
	I	P	**L**	**R**	T	S	I	A	A	23 (17.6)
	I	P	F	S	T	S	I	**G**	A	
	I	P	F	S	T	**N/T**	I	**G**	A	
	I	P	F	**R**	T	**N/T**	I	**G**	A	
**3**	I	P	F	S	T	S	I	A	A	
	I	P	F	S	T	**N/T**	I	A	A	
	I	P	F	**R**	T	**N/T**	I	A	A	
	I	P	**L**	**R**	T	S	I	A	A	
	I	P	F	S	T	S	I	**G**	A	
	I	P	F	S	T	**N/T**	I	**G**	A	68 (51.9)
	I	P	F	**R**	T	**N/T**	I	**G**	A	
	I	P	F	**R**	T	**N/T**	I	**G**	**G**	
**4**	I	P	F	S	T	**N/T**	I	A	A	3 (2.3)
	I	P	F	**R**	T	**N/T**	I	A	A	
**5**	I	P	F	S	T	S	I	A	A	
	I	P	F	S	T	**N/T**	I	A	A	
	I	P	F	**R**	T	S	I	A	A	
	I	P	F	**R**	T	**N/T**	I	A	A	14 (10.7)
	I	P	F	S	T	S	I	**G**	A	
	I	P	F	**R**	T	S	I	**G**	A	
**6**	I	P	F	S	T	**N/T**	I	A	A	3 (2.3)
	I	P	F	**R**	T	**N/T**	I	**G**	A	

Single mutation at codon position 661**V** and 651**D** in repeat region resulted in generation of novel repeat types 1b, 2d and 3c, respectively. TRV Type 1a, 2b, 3a, 3c, 5a was observed in all sampling sites. Type 4c in Baluchistan and Type 2a, 2c, 2d, 4a, 4b and 6a were observed exclusively in Baluchistan and Karachi isolates only.

## Discussion

This is the first study to provide baseline molecular epidemiological data on prevalence of SP resistance associated SNPs in *P. vivax* clinical isolates from southern Pakistan. In *dhfr,* wild type, single and double mutant alleles were observed in frequencies of 38.9, 19.8 and 27.4% respectively, indicating that wild type and double mutants are prevalent in this region. At PYR interaction site, 117**N** and 58**R** were found to be prevalent, both as a single mutant as well as in combination with other non-synonymous mutations while 57**L** mutation was detected in combination with 58**R** in low frequency (2.29%). These findings are similar to those reported from Khyber Pukhtunkhwa (KPK) and Bannu district of Pakistan [6,15], India [11-13,21], Iran [22], Afghanistan [14], China [23], Madagascar [24], East Timor [25], Thailand [19,26-28], Indonesia [29,30] and Mauritania [8]. The *dhfr* resistance patterns, commonly observed worldwide, of primary accumulation of mutations in codon 117 and 58 followed by amino acid change in codon 57 [19] was also observed in both northern and southern Pakistan, indicating a progressive tolerance in *P. vivax* to SP. *In vitro* studies have shown that compared to wild type enzymes, 58**R**, 117**N** and 57**L** mutant enzymes show approximately 1.9 to >500-fold increase in IC_50_ when tested against PYR and chlorcycloguanil [29,31]. Corroboration of this finding with clinical data has also shown association of these mutations with treatment failure cases [24,30]. Therefore, findings from this study indicate that in Pakistan, SP used for *P. falciparum* infection is selecting SP mutant alleles in *P. vivax* isolates. Consequently, extensive transmission of these resistant alleles within the population may affect SP usage for *Plasmodium* species in this region.

A distinctive finding, with respect to Pakistan, was the detection of serine to threonine (S-T) mutation at 117 codon position. This mutation has not previously been reported from other areas of Pakistan [6,15]. Globally, 117**T** substitution has been reported in areas of high transmission intensity and SP pressure, such as MaeSot Thailand [27], Myanmar [32], Vietnam [33], Indonesia [30], India [11,12]. Studies have shown that similar to 58**R**,117**N** and 57**L** mutation, presence of 117**T** mutation also increases IC_50_ of yeast expression system ~40 and ~43-fold. Sequential addition of non-synonymous mutations further increases the IC_50_ as high as >500-fold compared to WT [29,31]. Clinically, patients exhibiting 117**T** allele have also been found to be highly resistant to SP treatment [30]. Thus, this mutation is associated significantly with extensive drug resistance and high SP pressure in an area. In this study, 117**T** allele was isolated from Karachi as a double and quadruple mutant in combination of 58**R**/117**T**, 117**T**/119**K** and 58**R**/117**T**/114**R**/119**K** indicating that *P. vivax* isolates in this area may be exposed to SP pressure, resulting in selection of *dhfr* alleles associated with treatment failure. Though observed in low frequency, natural positive selection of this mutation requires serious consideration as this may have significant impact on SP treatment in this area.

Another unique finding in this study was the detection of 50**I** allele, which is being reported for the first time in southern Pakistan. Sequencing analysis of the respective allele resulted in the retrieval of two sequences from Pakistan exhibiting similar mutation (accession number JN 794518 and JN 794520). This finding may be significant since presence of 50**I**, reconfirmed in isolates from other areas of Pakistan, corroborates findings from this study. The significance of this mutation is that codon N50 in *P. vivax* WT corresponds with N51 codon of *P. falciparum* WT *dhfr* domain [31]. Previous studies, carried out via detection of drug-resistant alleles and *in vitro* culturing in *P. falciparum,* have reported that mutation in codon 51 from asparagine-isoleucine (N-**I**), in combination with other non-synonymous mutations, particularly 108**N**, is associated with high-level PYR resistance [12,34]. Studies on similar amino acid change in *P. vivax*, N50**I**, in combination with 117**N** allele (equivalent to 108**N** in *P. falciparum*) have been shown to increase the IC_50_ of yeast expression system up to ~57-fold [31]. Therefore, presence of this particular allele in Pakistan indicates that *P. vivax* isolates are selecting distinct alleles that may have a role in drug resistance. However, the effect of this mutation on drug resistance could only be assessed by *in vitro* culturing or in silico using homology modelling and molecular docking. Data from this study was not analysed using these tools and therefore significance of this mutation in drug resistance cannot be commented upon.

Non-synonymous mutations 114**R**, 119**K** and synonymous mutation, 69Y was predominantly, observed in Karachi samples. The 69Y mutation has been reported from high transmission areas of India [11,13] and therefore indicates natural selection of this allele in two neighbouring countries. However, since it is a synonymous mutation, its role in drug resistance is not implied.

In *dhps,* 383**G** and 553**G** alleles were observed in combination with *dhfr* 117**N/T** and 58**R** alleles. The result for 383**G** is in accordance with published data from India [11], Thailand [27], Madagascar [24], Pakistan [6] and Afghanistan [14]. However, the 553**G** haplotypes is being reported for the first time from Pakistan. Previously, this combination has been reported from high transmission area of India [11]. However, since this mutant allele was observed in low frequency in this study, analysis with respect to transmission is not possible.

Ten non-synonymous mutations were also observed in *dhps*, either alone or in combination with 383**G**. Of these, 459**A** and 601**I** has been reported from India [13]. Homology modelling and drug docking analysis in Indian isolates showed no apparent change in the efficacy of drug binding due to the presence of these mutations. Though, this conclusion could be extrapolated for this study’s samples, the difference is the occurrence of these mutations in combination with 383**G** and other non-synonymous mutations. Furthermore, a majority of these mutations was observed near the SPX interaction site (373**T**, 380**K**, 389**T**, 392**D**, 393**P**) and, therefore, the combined effect of these mutations on drug binding needs to be analysed before any conclusion can be drawn.

Non-synonymous mutations at 651**D** and 661**V** have been observed for the first time in isolates from Pakistan. Since these mutations were falling in the repeat region, novel TRV types were generated (Table 4). Mutations in the repeat regions are thought to have little impact on drug binding, therefore their role in drug susceptibility could be ruled out.

The high diversity observed in *dhps* in Pakistan is a matter of concern with respect to SP usage. However, an additional factor that may be influencing the selection of *dhps* resistant alleles is use of co-trimethoxazole (Trimethoprim + Sulphamethoxazole). This drug is commonly used in Pakistan for the treatment of bacterial infections or for prophylaxis and, therefore, sulpha drug pressure intrinsically exists in the environment [16]. In *P. falciparum*, complete cross resistance has been reported among sulpha drugs with variety of *dhps* genotypes [35,36]. It is possible that similar cross-resistance may be influencing the selection of resistant alleles by exerting pressure on *P. vivax* genes.

Studies have shown that TRV observed in both *dhfr* and *dhps* can be clustered as genotypes according to their distribution in a particular area [11]. TRV Type B in *dhfr* and Type 3a in *dhps* would thus be considered as prevalent genotypes in southern Pakistan. TRV Type 6a, having 11 repeats was observed in three patients from Karachi, all exhibiting severe disease symptoms. Similar finding was reported from India [13] and it could be assumed, though preliminarily, that Type 6a may be influencing the immunological response during vivax infection, possibly leading to disease severity. However, since this is novel data, based on sequencing only, serological studies need to be conducted before association between TRV types and disease severity can be implied.

Analysis of TRV association with mutation revealed that the predominant Type B and Type 3a were commonly associated with both WT and mutant alleles. These observations speculate that *dhfr* and *dhps* mutant alleles carrying the respective tandem repeat variant types could be susceptible to development of SP resistance associated mutations. Usefulness of tandem repeat variant types as molecular markers to predict the risk of resistance development has been implied in some studies [11,14], and in vitro studies would be able to provide a better understanding of the respective association and their impact on infection dynamics.

The results from this study show that diverse drug-resistant alleles of *P. vivax* are circulating in southern Pakistan especially in Karachi. Karachi is the largest and most populous metropolitan city of Pakistan with a population of 23.5 million [37]. Being a financial hub of Pakistan, extensive human movement is common in this area. Furthermore, during recent years, disaster induced displacement of individuals due to conflicts in northern Pakistan, floods and earthquakes have resulted in an increased net influx of migrants in Karachi [38]. This migration pattern is significant as malaria is spread by humans and this is particularly true for *P. vivax*, since hypnozoite acquired in one region could relapse months or years afterwards in a completely different place. It is possible that this large ongoing net influx of migrants may serve as parasite reservoirs carrying genetically diverse and drug resistant strains of *P. vivax* thus impacting malaria transmission in this area.

It is underlined that the aim of the study was to provide molecular epidemiological data on frequency distribution of *pvdhfr-pvdhps* haplotypes so that data on prevalent SP resistant alleles could be reported. Neither outcomes nor quantification of SP pressure was measured in this study and data interpretation was based on *in vitro* or findings reported in earlier studies on *P. vivax* isolates from other geographic areas. However, similarity of these results with those reported from other countries allows a generalized observation that SP pressure due to co-existence of *P. falciparum* exists in southern Pakistan, resulting in selection of common and distinct SP resistant alleles in *P. vivax*.

## Conclusion

The results indicate that in southern Pakistan *P. vivax* isolates are under natural selective pressure to accumulate SP resistance associated mutations. Therefore, urgent steps are required to monitor surveillance of drug resistance in Pakistan by concerned authorities.

## Abbreviations

P. vivax: *Plasmodium vivax*; P. falciparum: *Plasmodium falciparum*; Pvdhfr: *Plasmodium vivax dihydrofolate reductase*; pvdhps: *Plasmodium vivax dihydroptereoate synthase*; SPX: Sulphadoxine; PYR: Pyrimethamine; SP: Sulphadoxine-pyrimethamine; CQ: Chloroquine; PCR: Polymerase chain reaction; SNP: Single Nucleotide Polymorphisms; WT: Wild type; SM: Single mutation; DM: Double mutation; TM: Triple mutation; QM: Quadruple mutation; TRV: Tandem repeat variant; WHO: World Health Organization; IC50: Minimal inhibitory concentration.

## Competing interests

The authors declare that they have no competing interests.

## Authors’ contributions

AR performed PCR genotyping, data entry, statistical analysis and interpretation as well composed the manuscript. NKG designed, planned the study and reviewed the final draft. SK performed interpretation of sequencing results. MAB designed and planned the study, performed data analysis and interpretation and wrote the report. All authors read and approved the final manuscript.
